# A Founder Effect Led Early SARS-CoV-2 Transmission in Spain

**DOI:** 10.1128/JVI.01583-20

**Published:** 2021-01-13

**Authors:** Francisco Díez-Fuertes, María Iglesias-Caballero, Javier García-Pérez, Sara Monzón, Pilar Jiménez, Sarai Varona, Isabel Cuesta, Ángel Zaballos, Mercedes Jiménez, Laura Checa, Francisco Pozo, Mayte Pérez-Olmeda, Michael M. Thomson, José Alcamí, Inmaculada Casas

**Affiliations:** a AIDS Immunopathology Unit, National Center of Microbiology, Instituto de Salud Carlos III, Majadahonda, Madrid, Spain; b IDIBAPS–Hospital Clinic de Barcelona, Barcelona, Spain; c Respiratory Virus and Influenza Unit, National Center of Microbiology, National Influenza Center, Instituto de Salud Carlos III, Majadahonda, Madrid, Spain; d Bioinformatics Unit, Instituto de Salud Carlos III, Majadahonda, Madrid, Spain; e Genomics Unit, Instituto de Salud Carlos III, Majadahonda, Madrid, Spain; f HIV Biology and Variability Unit, National Center of Microbiology, Instituto de Salud Carlos III, Majadahonda, Madrid, Spain; University of California, Irvine

**Keywords:** COVID-19, Europe, SARS-CoV-2, Spain, phylodynamics, phylogeography

## Abstract

Severe acute respiratory syndrome coronavirus 2 (SARS-CoV-2) whole-genome analysis has identified five large clades worldwide which emerged in 2019 (19A and 19B) and in 2020 (20A, 20B, and 20C). This study aimed to analyze the diffusion of SARS-CoV-2 in Spain using maximum-likelihood phylogenetic and Bayesian phylodynamic analyses. The most recent common ancestor (MRCA) of the SARS-CoV-2 pandemic was estimated to have emerged in Wuhan, China, around 24 November 2019. Phylogenetic analyses of the first 12,511 SARS-CoV-2 whole-genome sequences obtained worldwide, including 290 from 11 different regions of Spain, revealed 62 independent introductions of the virus in the country. Most sequences from Spain were distributed in clades characterized by a D614G substitution in the S gene (20A, 20B, and 20C) and an L84S substitution in ORF8 (19B) with 163 and 118 sequences, respectively, with the remaining sequences branching in 19A. A total of 110 (38%) sequences from Spain grouped in four different monophyletic clusters of clade 20A (20A-Sp1 and 20A-Sp2) and 19B clade (19B-Sp1 and 19B-Sp2) along with sequences from 29 countries worldwide. The MRCAs of clusters 19A-Sp1, 20A-Sp1, 19A-Sp2, and 20A-Sp2 were estimated to have occurred in Spain around 21 and 29 January and 6 and 17 February 2020, respectively. The prevalence of clade 19B in Spain (40%) was by far higher than in any other European country during the first weeks of the epidemic, probably as a result of a founder effect. However, this variant was replaced by G614-bearing viruses in April. *In vitro* assays showed an enhanced infectivity of pseudotyped virions displaying the G614 substitution compared with those having D614, suggesting a fitness advantage of D614G.

**IMPORTANCE** Multiple SARS-CoV-2 introductions have been detected in Spain, and at least four resulted in the emergence of locally transmitted clusters that originated not later than mid-February, with further dissemination to many other countries around the world, and a few weeks before the explosion of COVID-19 cases detected in Spain during the first week of March. The majority of the earliest variants detected in Spain branched in the clade 19B (D614 viruses), which was the most prevalent clade during the first weeks of March, pointing to a founder effect. However, from mid-March to June 2020, G614-bearing viruses (clades 20A, 20B, and 20C) overcame D614 variants in Spain, probably as a consequence of an evolutionary advantage of this substitution in the spike protein. A higher infectivity of G614-bearing viruses than D614 variants was detected, suggesting that this substitution in SARS-CoV-2 spike protein could be behind the variant shift observed in Spain.

## Abstract (precis)

Multiple SARS-CoV-2 introductions have been detected in Spain, and at least four resulted in the emergence of locally transmitted clusters that originated not later than mid-February, with further dissemination to many other countries around the world, and a few weeks before the explosion of COVID-19 cases detected in Spain during the first week of March. The majority of the earliest variants detected in Spain branched in the clade 19B (D614 viruses), which was the most prevalent clade during the first weeks of March, pointing to a founder effect. However, from mid-March to June 2020, G614-bearing viruses (clades 20A, 20B, and 20C) overcame D614 variants in Spain, probably as a consequence of an evolutionary advantage of this substitution in the spike protein. A higher infectivity of G614-bearing viruses than D614 variants was detected, suggesting that this substitution in SARS-CoV-2 spike protein could be behind the variant shift observed in Spain.

## INTRODUCTION

The symptom onset of the first case of coronavirus disease 2019 (COVID-19) was reported on 1 December 2019 in the city of Wuhan, China, and the initial outbreak was related to the Huanan seafood market (1–3). Metagenomic RNA sequencing from these first cases identified a new RNA virus initially designated 2019-nCoV (4). This virus was subsequently named severe acute respiratory syndrome coronavirus 2 (SARS-CoV-2), the seventh representative of *Coronaviridae* with the capacity to infect humans (4). The World Health Organization (WHO) described COVID-19 as a pandemic on 11 March 2020, after more than 118,000 cases in 114 countries and 4,291 deaths (5). Phylogenetic analyses have confirmed the taxonomic classification of SARS-CoV-2 as a new member of the genus *Betacoronavirus* (subgenus *Sarbecovirus*) (2). SARS-CoV-2 was found to be sufficiently similar to SARS-CoV to be also considered a new member of the same species that caused the 2002–2003 epidemic (4). Two coronaviruses isolated from two species of horseshoe bats in the province of Yunnan, China, are the closest relatives of SARS-CoV-2 found so far: RaTG13, isolated from Rhinolophus affinis in 2013, and RmYN02, isolated from Rhinolophus malayanus in 2019 (1, 6, 7). However, other SARS-CoV-2-related coronaviruses have recently been isolated from pangolins (Manis javanica), showing less overall sequence identity within the S gene but the same residues which are key for ACE2 binding and human infection capacity within the receptor binding domain (RBD) of SARS-CoV-2 (8). However, some other differences have been observed between SARS-CoV-2 and pangolin coronaviruses, leaving unresolved the question about the identification of a possible intermediate host of the new virus (6, 9).

SARS-CoV-2 infections have been reported in all countries from Europe, causing hundreds to thousands of deaths in almost all of them (10). According to the open-source project Nextstrain, five larger clades of SARS-CoV-2 have been identified so far. Two emerged in 2019: 19A, which is considered the root clade, and 19B, marked by substitutions C8782T and T28144C. Three other clades appeared in 2020: 20A, distinguished from 19A by the substitutions C3037T, C14408T, and A23403G; 20B, characterized by three consecutive substitutions (G28881A, G28882A, and G28883C); and 20C, distinguished by the substitutions C1059T and G25563T (11). The first clades, 19A and 19B, were both prevalent during the first months of the outbreak in Asia, whereas 20A comprised mainly sequences from Europe in early 2020. Clades 20B, mainly comprising sequences from Europe, and 20C, a North American clade, also appeared in 2020 (11).

According to Nextstrain, almost all sequences in clade 19B are characterized by the presence of L84S in ORF8 (GISAID clade S), whereas the clades that emerged in 2020 (20A, 20B, and 20C) are characterized by D614G in the S protein (GISAID clade G) (10). The situation in Europe seems to be mainly dominated by clades 20A and 20B, although some countries, like Spain, showed a peculiar high prevalence of clade 19B. The increasing number of whole-genome sequences from viruses collected in Europe allows the performance of phylogenetic and evolutionary analyses to better understand the transmission dynamics within the most affected countries in Europe.

According to the European Centre for Disease Prevention and Control (ECDC), the first cases of SARS-CoV-2 infections in Europe were reported in week 5, 26 January to 1 February 2020, with 5 cases in France (one locally transmitted), 4 in Germany (all locally acquired), two imported cases in Italy, two imported cases in the United Kingdom, and one imported case in Finland (12). In Spain, the first two cases were reported during week 6, 2 to 8 February, the first in La Gomera (Canary Islands), related to a known cluster in Germany, and the second in Mallorca (Balearic Islands), in a British citizen who was initially in contact with a confirmed positive patient in France who returned from Singapore (13, 14). The next 5 reported cases in Spain occurred in patients with a history of travel from Italy and were detected before 26 February in Tenerife (Canary Islands), Catalonia, Castellón (Valencia region), and Madrid (15). In 2 days, Spain reported 18 new cases in different regions: Canary Islands, Catalonia, Andalusia, and Valencia. In week 10, 1 to 7 March 2020, a total of 261 confirmed positive cases were reported in the entire country (16). The increasing number of whole-genome sequences of SARS-CoV-2 circulating in Europe allows the performance of phylodynamic studies to better understand the transmission dynamics of the virus in Spain and in Europe and ideally to evaluate the capacities of respiratory viral surveillance systems.

## RESULTS

### Phylogeny of SARS-CoV-2 samples from Spain.

**FIG 1:**
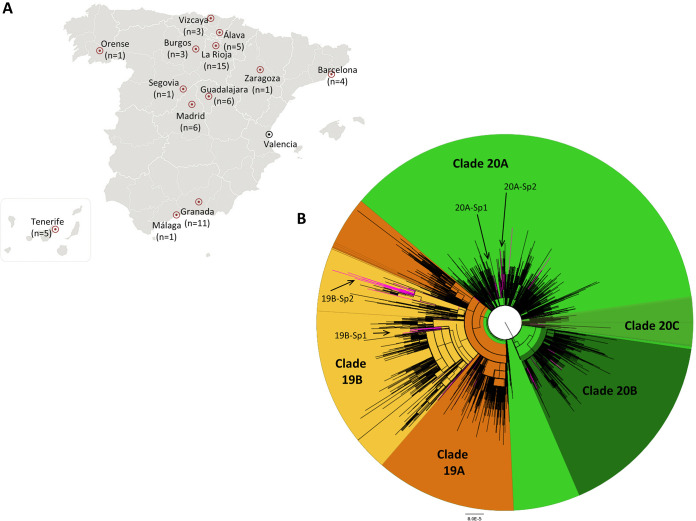
Origin and phylogeny of SARS-CoV-2 sequences from Spain. (A) Whole-genome sequencing of 61 samples from different locations (red circles) was carried out, and 229 other sequences from Valencia, Madrid, and Barcelona were retrieved from GISAID. (B) Phylogenetic tree of SARS-CoV-2 whole-genome sequences (*n* = 12,511) inferred by FastTree software v2.1.11 using a generalized time-reversible (GTR) model of nucleotide evolution. Sequences included in clades that emerged in 2019 (orange) and 2020 (green) are differentiated. The sequences from Spain are highlighted in pink to differentiate the 62 independent introductions observed in the country. The four monophyletic clusters with a probable origin in Spain (19B-Sp1, 19B-Sp2, 20A-Sp1, and 20A-Sp2) are indicated.

The mean depth of coverage of the 61 samples sequenced was 7,324×, with values ranging from 227× to 82,221×. Approximately 95% of the genome sequences reached a depth of coverage greater than 20× (68% to 100%). A total of 290 whole-genome sequences were obtained from respiratory samples studied in 11 different Spanish regions (Fig. 1). These sequences branched in the five major phylogenetic clusters defined by Nextstrain. A total of 154 (53.1%) harbored the D614G substitution in the S protein and were distributed in clades 20A (*n* = 126; 43.4%), 20B (*n* = 23; 7.9%), and 20C (*n* = 5; 1.7%). On the other hand, 114 (39.3%) branched in clade 19B, characterized by the presence of the L84S substitution in ORF8. The remaining 23 genome sequences branched within clade 19A (7.9%), including 13 sequences presenting the G251V substitution in ORF3 (4.5%) and 10 without this genetic marker (3.4%) (Table S1).

Four monophyletic clusters including more than 8 sequences from at least two Spanish cities were identified, two within clade 20A (20A-Sp1 and 20A-Sp2) and the other two within clade 19B (19B-Sp1 and 19B-Sp2); cluster 20A-Sp1 comprised 63 sequences from 14 different countries, including 17 from Spain; cluster 20A-Sp2 comprised 70 sequences from 15 countries, 9 of them from Spain; cluster 19B-Sp1 comprised 64 sequences from 12 countries, including 32 from Spain; and finally, cluster 19B-Sp2 comprised 84 genome sequences from 16 countries, including 52 from Spain.

### Global expansion of SARS-CoV-2 in Europe.

The most recent common ancestor (MRCA) estimated for the data set built with sequences from Wuhan and from the first reporting European countries was located in Wuhan (posterior probability [PP] = 0.99) around 24 November 2019 (95% highest posterior density [HPD] interval from 30 October to 17 December 2019). Bayesian stochastic search variable selection (BSSVS) analysis revealed highly probable diffusions from Wuhan to all the European countries included in the data set, i.e., England (Bayes factor [BF] > 100; PP = 1.00), Germany (BF > 100; PP = 1.00), France (BF > 100; PP = 1.00), Spain (BF > 100; PP = 1.00), Finland (BF > 100; PP = 0.99), and Italy (BF = 29.1; PP = 0.93) (Fig. 1). This data set was also used to estimate the rate of evolution of SARS-CoV-2, which was 1.47 × 10^−3^ substitutions per site per year (95% HPD interval 1.08 × 10^−3^ to 1.87 × 10^−3^).

### Local transmission of clade 19B in Spain.

Approximately 40% of all genome sequences from Spain harbored the L84S substitution in ORF8, which is, by far, the highest frequency found in Europe. The proportion of clade 19B in other European countries (Belgium, Denmark, England, Finland, France, Italy, Norway, Sweden, Switzerland, and Wales) was less than 1%. Countries with higher percentages of S84 in ORF8 were Greece (8%), Scotland (4%), Germany (3%), Iceland (3%), Portugal (3%), and Luxembourg (2%).

**FIG 2:**
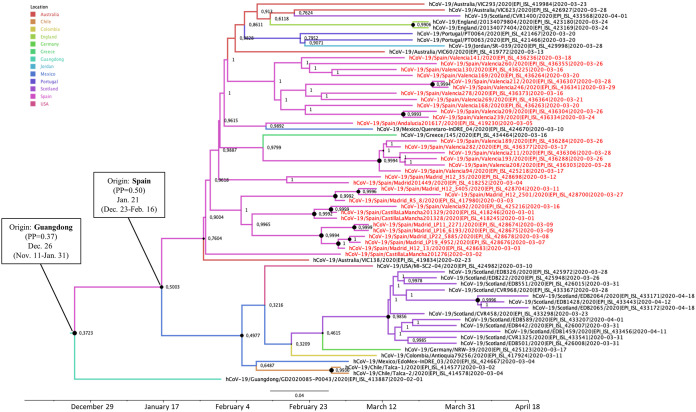
Maximum clade credibility (MCC) tree of cluster 19B-Sp1. Branch colors indicate the most probable location of the MRCA, and node labels indicate the posterior probability supporting the estimated MRCA location. Node support values are indicated by node size (only nodes with PP of ≥0.8 are considered well supported). The scale axis represents estimated dating of the MRCA for each cluster, and label spacing defines exactly 9.16 days from the most recent sample included in the analysis.

The most probable geographic location of the MRCA of the two phylogenetic clusters branching in 19B clade (19B-Sp1 and 19B-Sp2) was Spain, with posterior probabilities of 0.50 and 0.56, respectively. The date of the MRCA of 19B-Sp1 was estimated around 21 January (95% HPD interval, 23 December to 16 February 2020) and included sequences from the Valencia, Andalusia, Madrid, and Castilla-La Mancha regions, clustered with sequences from Australia, Chile, Colombia, England, Germany, Greece, China, Jordan, Mexico, Portugal, Scotland, and the United States (Fig. 2). A probable direct diffusion between countries was detected only from Spain to Germany (BF = 32.8; *P* = 0.86).

**FIG 3:**
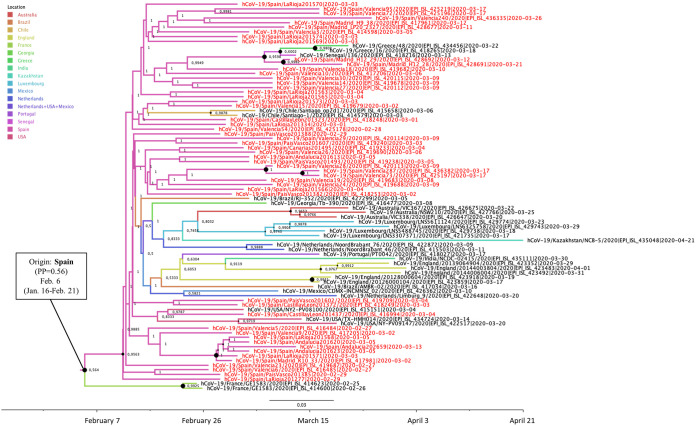
Maximum clade credibility (MCC) tree of cluster 19B-Sp2. Branch colors indicate the most probable location of the MRCA, and node labels indicate the posterior probability supporting the estimated MRCA location. Node support values are indicated by node size (only nodes with PP of ≥0.8 are considered well supported). The scale axis represents estimated dating of the MRCA for each cluster, and label spacing defines exactly 9.16 days from the most recent sample included in the analysis.

Cluster 19B-Sp2 included sequences from La Rioja, Valencia, Madrid, Castilla y León, Basque Country, Canary Islands, and Andalusia and clustered with 84 sequences from 15 other countries (Australia, Brazil, Chile, England, France, Georgia, Greece, India, Kazakhstan, Luxembourg, Mexico, Netherlands, Portugal, Senegal, and the United States). The date of the MRCA of 19B-Sp2 cluster was estimated around 6 February 2020 (95% HPD interval, 16 January to 21 February 2020) (Fig. 3). Regarding cluster 19B-Sp2, probable diffusions from Spain to other countries were not found, but diffusions from Greece to Portugal (BF = 27.0; *P* = 0.80) and from Portugal to India (BF= 39.13; *P* = 0.85) were identified.

### Local transmission of clade 20A in Spain.

The frequency of the G614 substitution in Spanish genomes was the lowest observed compared to other European countries (52.9% versus >70%), including Austria, Belgium, Denmark, Finland, France, Germany, Greece, Iceland, Italy, Luxembourg, Portugal, Scotland, Sweden, and Switzerland. Countries with intermediate frequencies were England (64%), Netherlands (63%), and Wales (61%).

**FIG 4:**
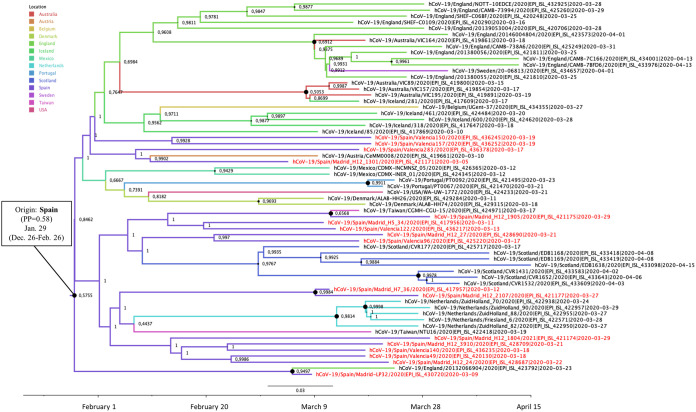
Maximum clade credibility (MCC) tree of cluster 20A-Sp1. Branch colors indicate the most probable location of the MRCA, and node labels indicate the posterior probability supporting the estimated MRCA location. Node support values are indicated by node size (only nodes with PP of ≥0.8 are considered well supported). The scale axis represents estimated dating of the MRCA for each cluster, and label spacing defines exactly 9.16 days from the most recent sample included in the analysis.

The MRCAs of the two phylogenetic clusters branching in the 20A clade (20A-Sp1 and 20A-Sp2) were located in Spain, and both included sequences from Madrid and Valencia. Cluster 20A-Sp1 comprised 63 sequences from 13 other countries (Australia, Austria, Belgium, Denmark, England, Iceland, Mexico, Netherlands, Portugal, Scotland, Sweden, Taiwan, and the United States) (Fig. 4). The MRCA of cluster 20A-Sp1 was estimated in Spain (PP = 0.58) around 29 January (95% HPD interval, 26 December 2019 to 26 February 2020). Several diffusion pathways between countries were identified from Spain to Portugal (BF = 20.66; *P* = 0.79), from Spain to Mexico (BF = 13.06; *P* = 0.70), from Mexico to Belgium (BF = 72.29; *P* = 0.93), and from Belgium to the United States (BF = 81.52; *P* = 0.93).

**FIG 5:**
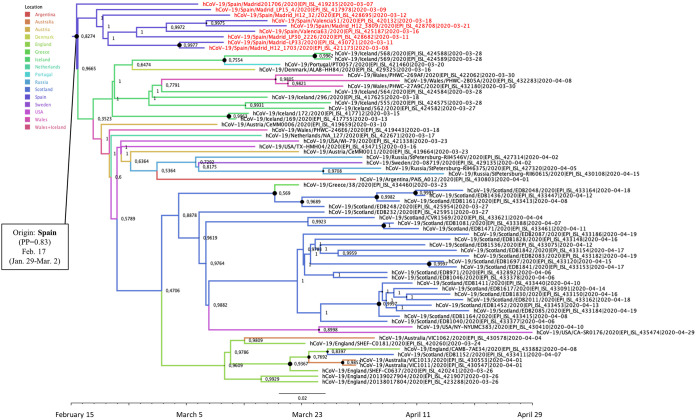
Maximum clade credibility (MCC) tree of cluster 20A-Sp2. Branch colors indicate the most probable location of the MRCA, and node labels indicate the posterior probability supporting the estimated MRCA location. Node support values are indicated by node size (only nodes with PP of ≥0.8 are considered well supported). The scale axis represents estimated dating of the MRCA for each cluster, and label spacing defines exactly 9.16 days from the most recent sample included in the analysis.

Cluster 20A-Sp2 comprised 70 sequences from 14 other countries (Argentina, Australia, Austria, Denmark, England, Greece, Iceland, Netherlands, Portugal, Russia, Scotland, Sweden, the United States, and Wales). The MRCA of cluster 20A-Sp2 was estimated in Spain (PP = 0.83) around 17 February (95% HPD interval, 29 January to March 2020) (Fig. 5). Direct diffusions from Spain to other countries were not identified; however, diffusions from Iceland to Austria (BF = 74.85; *P* = 0.92) and from Austria to Denmark (BF = 13.02; *P* = 0.68) were well supported. Diffusions from Scotland and from Wales to Sweden were also identified (BF = 15.51 [*P* = 0.72] and BF = 12.73 [*P* = 0.67], respectively).

### Clade 19A in Spain.

Several sequences from Spain were distributed throughout clade 19A, which includes 13 Spanish sequences harboring the G251V substitution in ORF3 (GISAID clade V), 7 genomes included in GISAID group O, and 3 other sequences in GISAID group L.

**FIG 6:**
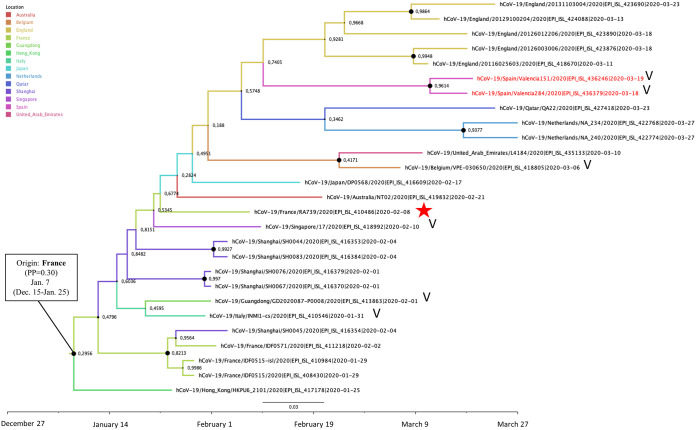
Maximum clade credibility (MCC) tree of the cluster including one sequence of the Contamines-Monjoie transmission chain (EPI-ISL-410486). Branch colors indicate the most probable location of the MRCA, and node labels indicate the posterior probability supporting the estimated MRCA location. Node support values are indicated by node size (only nodes with PP of ≥0.8 are considered well supported). The scale axis represents estimated dating of the MRCA for each cluster, and label spacing defines exactly 9.16 days from the most recent sample included in the analysis. The red star indicates the sequence associated with the Contamines-Monjoie cluster, and “V” indicates the presence of the G251V mutation in ORF3a protein.

The EPI-ISL-410486 sequence of the Contamines-Monjoie cluster in France branched in a phylogenetic cluster comprising 27 sequences from England, France, Qatar, Netherlands, United Arab Emirates, Belgium, Japan, Australia, Singapore, China (Shanghai and Guangdong), and Hong Kong and also two sequences from Spain (EPI-ISL-436246 and EPI-ISL-436379). The MRCA of this cluster was dated around 7 January (95% HPD interval, 15 December 2019 to 25 January 2020), although the geographic location of the MRCA was uncertain among France (PP = 0.30), Shanghai (PP = 0.28), and Hong Kong (PP = 0.25). This phylogenetic cluster included sequences with the G251V substitution in ORF3 protein along with other sequences without any of the major clade-defining residues (Fig. 6).

### D614 variants in Spain.

**FIG 7:**
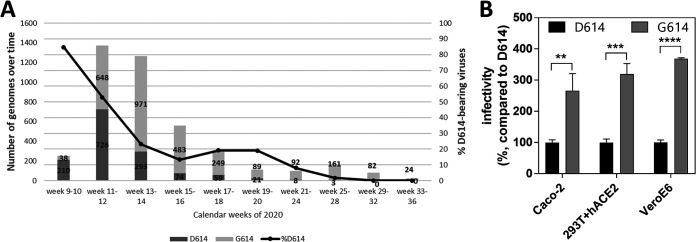
Founder effect of D614-variants in Spain and selective advantage of D614G substitution. (A) D614/G614 prevalence over time in Spain according to the data available on 8 July 2020. Increased infectivity of D614G pseudoviruses. VeroE6, Caco-2, and 293T cells expressing human ACE2 were infected with equal amounts (10 ng of Gag p24) of D614 or G614 pseudoviruses. Viral infectivity was determined by measuring luciferase activity in the cell lysates 48 h postinfection and is expressed as percent infectivity relative to that of D614 virus. Results are means and standard deviations for an experiment performed in triplicate. Results of a representative experiment out of three independent assays is shown. ****, *P* < 0.0001; ***, *P* < 0.001; **, *P* < 0.01 (unpaired two-tailed Student's *t* test).

By 6 March 2020, a total of 251 SARS-CoV-2 infections had been reported in Spain (16). According to GISAID, 175 genomes (69.7%) were obtained from samples collected in Spain before that date. Among these genomes, 30 (17.1%) carried the D614G substitution and were obtained from samples from nine different regions of Spain (Madrid, Andalusia, Canary Islands, Castilla y León, Extremadura, Catalonia, Balearic Islands, Basque Country, and Valencia). Among the remaining 145 samples carrying D614 (82.9%), the majority branched in the S clade (127; 72.6%) and especially within the A.2 lineage (64.6%), including samples from the regions where G614 viruses were detected plus La Rioja, Castilla La Mancha, and Aragón. These results, along with the high proportion of variants branching in clade 19B from samples collected during the first weeks of March, suggest that a founder effect drove the earliest transmissions in Spain (Fig. 7).

### Selective advantage of G614 variants *in vitro*.

Variants with the D614G substitution became dominant in Spain in week 12 (15 to 21 March 2020) (Fig. 7). To examine whether this fact could be explained by an evolutionary advantage, we quantified the infectious titers of pseudotyped lentiviral particles with either D614 or G614 SARS-CoV-2 spike protein in VeroE6, Caco-2, and HEK 293T cells stably expressing human ACE2 receptor. Viruses with G614 had significantly higher infectious titers than D614 variants, showing 3.7-, 3.2-, and 2.7-fold increases in VeroE6, 293T+hACE2, and Caco-2 cells, respectively (Fig. 7).

## DISCUSSION

SARS-CoV-2 infection is a major public health threat in Europe and worldwide. The disease caused by this virus (COVID-19) has emerged as a major cause of deaths in countries like Spain, where, as of the beginning of May 2020, more than 200,000 infections and around 25,000 deaths had been reported (17). Mitigation measures have been implemented in many countries, including Spain since 14 March, confining their populations at home to avoid new transmissions of the virus.

The global analysis of the origin of the ongoing pandemic of SARS-CoV-2 indicated that the time to the MRCA (tMRCA) was around 24 November 2019, with a 95% HPD interval from 30 October to 17 December 2019, in the city of Wuhan (PP = 0.99). This result is consistent with the epidemiological information about the first case of COVID-19 reported in Wuhan, which dated the symptom onset in this patient to 1 December 2019 (3). The estimated rate of evolution for SARS-CoV-2 was in the range of 1.08 × 10^−3^ to 1.87 × 10^−3^ substitutions per site per year, which is also comparable with the rates estimated for other epidemic coronaviruses, i.e., SARS-CoV (0.80 × 10^−3^ to 2.38 × 10^−3^) and Middle East respiratory syndrome coronavirus (MERS-CoV) (0.88 × 10^−3^ to 1.37 × 10^−3^) (18, 19). Taking into account the outbreak onset in Wuhan, it is worth pointing out that the city of Wuhan had direct flight connections with different European cities, including Paris (six weekly flights), London (three weekly flights), and Rome (three weekly flights) (20).

Many laboratories are urged to rapidly make genomic data of the virus publicly available. As more SARS-CoV-2 whole-genome sequences are generated, acquiring sequences of clades 20A and 20B in the European epidemic is of greater importance, especially clade 20A, which is the largest phylogenetic cluster so far in Europe. The earliest samples collected in Europe with genomic information were from France, Germany, Italy, England, and Finland at the end of January 2020 (23, 28, and 29 for the last three, respectively). The sequences from France (EPI-ISL-406596 and EPI-ISL-406597) and Italy (EPI-ISL-408068 and EPI-ISL-412974) were from patients infected in Wuhan and branched in clade 19A (GISAID clade V) (21). The case from Germany (EPI-ISL-406862) originated an outbreak after contact between a “healthy German businessman” and a Shanghai resident who tested positive for SARS-CoV-2 on 26 January (9). This cluster, branching in clade 20A, comprised at least 12 cases from Bavaria (13). On the other hand, the sequences from England (EPI-ISL-407071 and EPI-ISL-407073) branched in clade 19B, and the sequence from Finland (EPI-ISL-407079) branched in clade 19A (outside GISAID clade V). According to reports from the European Centre for Disease Prevention and Control (ECDC), these 5 countries (France, Italy, Germany, England, and Finland) were the first ones in which SARS-CoV-2 infections were detected in Europe (21). The results of the present study revealed that the diffusion of the virus from Wuhan to these five European countries and to Spain was highly probable. Thus, viruses branching in all the major clades prevalent at that time were already present in Europe at the end of January 2020.

Analyzing the information associated with the first 4,242 genome sequences indicates that the earliest samples collected in Spain were from the Canary Islands (EPI_ISL_539531, from Santa Cruz de Tenerife), Madrid (EPI-ISL-418251), Valencia (EPI-ISL-416483 and EPI-ISL-419689, from the city of Valencia), Castilla y León (EPI-ISL-418247, from Segovia), Extremadura (EPI_ISL_539557, from Badajoz), Balearic Islands (EPI_ISL_541968, from Palma de Mallorca), Andalusia (EPI-ISL-418243, from Granada, and EPI_ISL_455314, from Seville), Basque Country (EPI_ISL_455350, from Vitoria), and La Rioja (EPI_ISL_455336, from Logroño) from samples collected at the end of February 2020 (from 24 to 29) and branching in clades 20A (Canary Islands, Madrid, Castilla y León, and Extremadura), 20B (Canary and Balearic Islands and Valencia), 19B (Valencia, Andalusia, Basque Country, and La Rioja), and 19A (Andalusia). In other words, viruses branching in the four major clades that emerged in 2019 and early 2020 were detected in Spain at the end of February 2020. Regarding clade 20C, the first variant in Spain was detected in Andalusia (EPI_ISL_474922, from Málaga) on 3 March 2020. According to ECDC reports, a total of 25 SARS-CoV-2 cases were detected in Spain at the end of February, reaching 261 cases during the first week of March (15, 16). All this information, along with the phylogenetic analysis of the present study, suggests that multiple introductions of SARS-CoV-2 had occurred in Spain not later than the end of February and that representatives of all the major clades of the virus were already being detected in the first week of March.

Bayesian phylogenetic inference and coalescent-based population genetics were used to analyze different phylogenetic clusters, including samples from different regions of Spain, with the aim of elucidating their ancestry and spread in Spain and other countries. In this regard, four different phylogenetic clusters, including at least 8 samples collected in two or more cities in Spain, were further analyzed. Two of these clusters branched in clade 20A (20A-Sp1 and 20A-Sp2) and the other two branched in clade 19B (19B-Sp1 and 19B-Sp2). The most probable origin of these four clusters was Spain, suggesting that viruses belonging to them were more likely transmitted locally than imported from other countries. The MRCAs of 20A-Sp1 and 19B-Sp1 were dated around the last week of January 2020 (29 January 2020 for 20A-Sp1 and 21 January 2020 for 19-Sp1), whereas the origins of 20A-Sp2 and 19B-Sp2 were dated around 17 February 2020 and 6 February 2020, respectively. Moreover, these analyses suggested that these four clusters were disseminated to many other countries worldwide. 20A-Sp1 was probably exported to different countries in North America and Europe but also to Australia and Taiwan; 20A-Sp2 was spread mainly to other European countries but also to Argentina, Australia, and the United States; 19B-Sp1 was mainly disseminated to the Americas (Chile, Colombia, Mexico, and the United States) but also to China, Jordan, Australia, and different European countries; 19B-Sp2 was also spread to European countries, the Americas (Brazil, Chile, Mexico, and the United States), Asia (Georgia, India, and Kazakhstan), and Africa (Senegal). Therefore, it is possible that at least clusters 19B-Sp1 and 20A-Sp1 were already circulating in Spain in January 2020, since the 95% HPD intervals estimated for them were 23 December 2019 to 16 February 2020 and 26 December 2019 to 26 February 2020, respectively. In spite of these wide intervals and taking into account the most recent date of the 95% HPD interval estimated for 19B-Sp1, these results suggest that SARS-CoV-2 was already circulating in Spain through silent community transmission not later than mid-February 2020. This fact implies the presence of undocumented infections in Spain at least 3 weeks prior to the explosion of the 261 COVID-19 cases detected in the first week of March 2020 and a month before the country-wide lockdown went into effect on 14 March 2020. The presence of clade 19B viruses at the end of February to the beginning of March at different locations in Spain, including cities such as Madrid, Valencia, Seville, Granada, San Sebastián, Málaga, Guadalajara, Burgos, Vitoria, Bilbao, Logroño, and Santa Cruz de Tenerife, could point to a founder effect associated with the high prevalence of this clade observed in Spain in the following weeks. However, further analyses are necessary to discover the reasons for the high prevalence of clade 19B viruses in Spain compared with the rest of European countries. The high prevalence of genome sequences from Spain was also reported by Rambaut et al. (22) in their proposal about a new SARS-CoV-2 nomenclature, identifying the A.2 and A.5 lineages, in which clusters 19B-Sp2 and 19B-Sp1, respectively, are included.

The analysis of 69.7% of all the infections of SARS-CoV-2 reported in Spain by 6 March 2020 revealed a higher presence of D614 variants (82.9%) than G614 viruses (17.1%). However, this rate was later inverted, and G614 variants became the most prevalent in the country. Several studies have suggested that a selective advantage of G614 variants allowed these viruses to rapidly become dominant in the regions where they were introduced (23, 24). In the present study, an enhanced viral infectivity of G614 variants was observed in three different cell lines, suggesting that this substitution could alter SARS-CoV-2 transmissibility. A recent study also associated the D614G substitution with a decreased neutralization sensitivity to individual convalescent-phase sera (23). In spite of this selective advantage, D614 variants coexisted in Spain with G614-bearing viruses, reaching higher prevalence from the beginning of the epidemic until the end of March and suggesting that a founder effect of variants branching in 19B clade led the initial transmissions. However, by the end of March, G614 variants became the most prevalent in Spain, probably as a consequence of a fitness advantage compared with their D614 counterparts, observed in an *in vitro* assay with pseudotyped viruses. Similar results were reported by Korber et al. for Spain and other countries like the United Kingdom, Germany, and the United States, where the higher prevalences of D614 variants in initial stages were rapidly overcome by G614 variants (24). The fact that a good representation (69.7%) of the reported positive cases during the first weeks of the epidemic in Spain were sequenced suggests that the effect of sample sequencing bias is not influencing the observed decreasing tendency of D614 variants.

The main limitations of the present study are related to the fact that genomic sequences are being generated by diverse strategies following different steps that could affect the quality of the sequences. Different sample preparation techniques are being used, including overlapping amplicons, targeted capture where the viral RNA is enriched, and metagenomic total RNA sequencing of rRNA-depleted samples. The first two methods require less sequencing effort, but the possibility that some RNA molecules could be missed cannot be ruled out. In contrast, the metagenomic approach is hypothesis free, but it implies a high number of sequencing reads. Another point to take into account is the sequencing strategies *per se*, since several approaches are being used, including the use of Sanger sequencing and next-generation sequencing platforms, such as iSeq, MiSeq, NextSeq, and Novaseq from Illumina, MinION and GridION from Nanopore, and IonTorrent from Thermo Fisher (21). All these technologies also have their own biases. Finally, the informatics employed to analyze the data is the step where more diversity of options is being identified. For all these reasons, some of the genetic differences observed between samples could be attributable to the error rate of sequencing platforms, indicating that genomes may be more similar than observed. On the other hand, using a reference genome to align the reads instead of following a *de novo* approach could mask some real genetic differences. In this regard, initiatives in nf-core are trying to provide best-practice pipelines for the analysis of SARS-CoV-2 data in a peer-reviewed platform that includes some pipelines developed by the Bioinformatics Unit of the Instituto de Salud Carlos III (25–27).

No phylogenetic clusters associated with the initial imported cases from La Gomera and Mallorca (13, 14) have so far been identified as clusters with a MRCA located in Spain, suggesting that the local transmission of the virus was prevented or undetected. However, this study also revealed that the epidemic in Spain began as a consequence of sufficient silent introductions of viruses from different clades. In particular, four events of community transmission have been detected, and at least one of them, cluster 19B-Sp1 (a subcluster of the A.5 lineage described by Rambaut et al. [22]), was silently circulating in the country in mid-February and probably a few weeks earlier. These dates are several weeks before the wave of COVID-19 cases detected in the country in March 2020 and at least a month before the lockdown that went into effect on 16 March 2020. Therefore, it is essential to improve SARS-CoV-2 genomic surveillance in all regions of the country to avoid an underestimation of the genetic diversity of the virus circulating in Spain. Phylodynamic studies are crucial for understanding the dynamics of viral transmission, improving our knowledge about the origin and spread of SARS-CoV-2 worldwide and at the local level, and assessing the impact of intervention measures, such as country-wide lockdowns. Moreover, these analyses should also be considered for use in efforts to mitigate the pandemic, by evaluating the correct functioning of surveillance systems, identifying viral diffusion routes, and guiding response measures by public health authorities.

## MATERIALS AND METHODS

### Patient samples.

A total of 61 oropharyngeal and nasopharyngeal swabs from hospitalized patients in 13 different hospitals from Spain were obtained for genome sequencing (Fig. 1). These samples were collected from the last week of February to the first weeks of March 2020. The ages of these patients ranged from 18 to 95 years, with a mean value of 51.5 years; 62% were males and 38% were females.

### Whole-genome sequencing.

Two different approaches were performed in this work to obtain the whole-genome sequence of SARS-CoV-2. The first of these methods was a hybrid capture-based enrichment protocol. Clinical samples were processed and amplified by sequence-independent, single-primer-amplification reverse transcription-PCR (SISPA RT-PCR) as described in a previous work (28), and the preparation of libraries and probe hybridization reactions were performed according to the Twist SARS-CoV-2 research panel instructions (Twist Bioscience Corporation, CA, USA). The second approach employed was genome amplification using the ARTIC network’s PCR protocol based on the use of the pool of primers “ARTIC n-CoV-2019 v3” (29). In this case, libraries were constructed according to the Nextera XT library preparation kit instructions (Illumina, CA, USA). In both approaches, libraries were barcoded with nonoverlapping dual indexes, pooled, and sequenced using a MiSeq reagent kit (v2, 300 cycles; Illumina, CA, USA) in a MiSeq sequencer (Illumina, CA, USA).

### Viral consensus genome reconstruction.

Viral consensus genomes were obtained using a mapping against viral reference genome approach, followed by variant calling and consensus genome generation. This pipeline, called Viralrecon (https://github.com/nf-core/viralrecon), was written using Nextflow framework (https://www.nextflow.io/) in collaboration with the nf-core team (30). FastQC files containing raw reads were first analyzed for quality using FastQC v0.11.9 (http://www.bioinformatics.babraham.ac.uk/projects/fastqc/). Raw reads were trimmed for low-quality 3′ ends and adapter sequence removal using fastp v.0.20.1 (31). Trimmed reads were mapped against the reference SARS-Cov2 genome (NC_045512.2) with bowtie2 v.2.3.5.1 (32), and the mapping files coming from amplicon sequencing were trimmed with iVar v.1.2.2 (33) to remove amplicon primers. We used Picard v.2.22.8 (https://github.com/broadinstitute/picard) and SAMtools v.1.9 (34) to generate viral mapping stats. To obtain statistics about the host genome content, we performed kmer-based mapping of the trimmed reads against the GRCh38 NCBI human genome reference with Kraken2 v.2.0.9beta (35).

Variant calling was carried out using VarScan2 v.2.4.4 (36), from which we kept variants with an allele frequency higher than 80. Filtered variants were annotated using SnpEff v.4.5covid19 (37) and SnpSift v.4.3.1t (38). Finally, bedtools v2.29.2 (39) was used to obtain the viral genome consensus with the called variants and mask genomic regions with coverage values lower than 10×. Final summary reports were created using MultiQC v.1.9 (40).

### Data sets.

In order to investigate the transmission dynamics of SARS-CoV-2 in Spain, different data sets were retrieved from GISAID (21). First, all the whole-genome sequences (>29,000 bp) of the virus with high coverage from countries of all the continents were obtained (*n* = 12,511) to evaluate how the samples from Spain (*n* = 290) are distributed (including the samples sequenced in the present study). Then, another data set was analyzed including only whole-genome sequences from the countries in Europe which first reported positive cases and from Wuhan, the city of the outbreak onset, to study the dynamics of viral transmission from Wuhan to Europe. Sequences were aligned using progressive and iterative refinement methods depending on data size as implemented in MAFFT version 7 software, and sequences were manually edited using AliView v1.26 (41, 42). We followed the recommendations of De Maio et al. (43), from the European Bioinformatics Institute, for filtering and masking alignments of SARS-CoV-2 sequences. In brief, positions 1 to 55 and 29804 to 29903 (when aligned to the reference sequence with GenBank accession no. MN908947.3) and several highly homoplastic sites, mostly with no phylogenetic signal and/or low prevalence, were masked for the analyses (43). Finally, the first 4,242 genome sequences from Spain deposited in GISAID were used to investigate the prevalence of the D614G mutation throughout the epidemic.

### Phylogenetic and evolutionary analyses.

Phylogenies of large alignments were inferred by FastTree software v2.1.11 (44). Root-to-tip genetic distances against sample collection dates were measured with TempEst v1.5.1, and Bayesian time-scaled phylogenetic analyses were performed with BEAST v1.10.4 to estimate the date and location of the most recent common ancestors (MRCAs), as well as to estimate the rate of evolution of the virus (45, 46). BEAST priors were introduced with BEAUTi v1.10.4, including an uncorrelated relaxed molecular clock model with a lognormal rate distribution and the exponential growth coalescent model of population size and growth (45). The coalescent model was selected over other models because it assumes that a small random sample from a large population is included in the data set. Markov chain Monte Carlo (MCMC) runs of 100 million states sampling every 10,000 steps were computed. The convergence of MCMC chains was checked using Tracer v.1.7.1, ensuring that the effective sample size (ESS) values were greater than 200 for each estimated parameter (46). The maximum clade credibility (MCC) trees were obtained from the tree posterior distribution using TreeAnnotator after a 10% burn-in (45). Phylogenetic clusters including sequences from Spain were further analyzed to estimate the time and location of the most recent common ancestors. Phylogenetic trees were visualized and edited with FigTree v1.4.4 (47).

### Viral transmission dynamics.

Discrete trait evolutionary histories associated with phylogenies were analyzed with SpreaD3 (48). BEAST outputs were converted to a JavaScript Object Notation (JSON) and annotated phylogenies were projected on a map in geoJSON data structure format. Parsed input data were also converted to Keyhole Markup Language (KLM) for visualizations in virtual globe software like Google Earth. Well-supported rates between locations in the phylogeographic reconstructions were identified through Bayes factors by using the Bayesian stochastic search variable selection (BSSVS) procedure implemented in BEAST (49).

### Cells and SARS-CoV-2 S constructs.

Vero E6 (African green monkey kidney) and Caco-2 (human, colon) cell lines were kindly provided by A. Alcami (Consejo Superior de Investigaciones Científicas [CSIC], Madrid, Spain) and M. A. Muñoz (Hospital Gregorio Marañón, Madrid, Spain), respectively. HEK 293T cells stably expressing recombinant human ACE2 (HEK-hACE2 cells) were generated by transduction with the pLVX-hACE2 lentiviral vector (a gift from D. Lavillette, Shanghai, China) encoding the receptor sequence. All the above-mentioned cells were cultured in Dulbecco modified Eagle medium (DMEM) supplemented with 10% fetal calf serum (FCS), 2 mM l-glutamine, and 100 U/ml penicillin and streptomycin.

For the generation of the expression plasmids for SARS-Cov-2 S glycoprotein, codon-optimized cDNA (QHU36824.1) lacking the last 19 amino acids (50) was synthesized (GeneArt Gene Synthesis, Thermo Fisher Scientific), PCR amplified, and cloned into the pcDNA3.1 expression vector (Invitrogen). A mutant clone introducing the D614G change was created by site-directed mutagenesis. The DNA sequences of both S glycoproteins (D614 and G614) were confirmed by sequencing.

### SARS-CoV-2 S pseudovirions and infection assays.

NL4.3 pseudotypes were generated with the previously described plasmid pNL4-3ΔenvRen (51). Briefly, *Renilla* luciferase reporter pseudoviruses were prepared by cotransfecting pNL4-3ΔenvRen with each of the two S-encoding-plasmids in HEK 293T cells using the calcium phosphate method. Forty-eight hours posttransfection, cell culture supernatants were harvested, clarified by centrifugation at 500 × *g* for 5 min, and frozen at −80°C. The amount of p24 antigen in the supernatants was quantified by electrochemiluminescence immunoassay (Roche Diagnostic). Pseudovirus infectivity was assessed using a modified replication capacity assay (52). VeroE6, Caco-2, or hACE2-expressing HEK 293T cells were plated onto flat-bottom 96-well plates (5 × 10^4^ cells/well) and incubated overnight at 37°C under 5% CO_2_. Then, cells were infected with equal amounts of pseudoviruses as assessed by p24 measurement (10 ng p24 Gag/well). At 48 h postinfection, cells were lysed, and viral infectivity was assessed by measuring luciferase activity (*Renilla* luciferase assay; Promega, Madison, WI) using a 96-well-plate luminometer (Orion; Berthold, Oak Ridge, TN). Viral infectivities, expressed as percentages, were determined by comparing the luciferase activity induced by G614 pseudoviruses with the luciferase activity induced by D614 pseudoviruses. Graphs and statistical analyses were generated with Prism, version 7.0 (GraphPad Software, Inc.).

### Data availability.

All the genomic sequences generated and used in the present study have been deposited in GISAID (https://www.gisaid.org) and are fully accessible for registered users within the “browse” option of the EpiCoV database. The GISAID ID numbers of genomic sequences from Spain are in Table S1.

## Supplementary Material

Supplemental file 1

Supplemental file 2
