# Supplementary material for: A Founder Effect Led Early SARS-CoV-2 Transmission in Spain
Source: J Virol. 2021 Jan 13;95(3):e01583-20. doi: 10.1128/JVI.01583-20 (PMC7925114; doi:10.1128/JVI.01583-20)
Supplement: Supplemental file 1 [file JVI.01583-20-s0002.pdf]

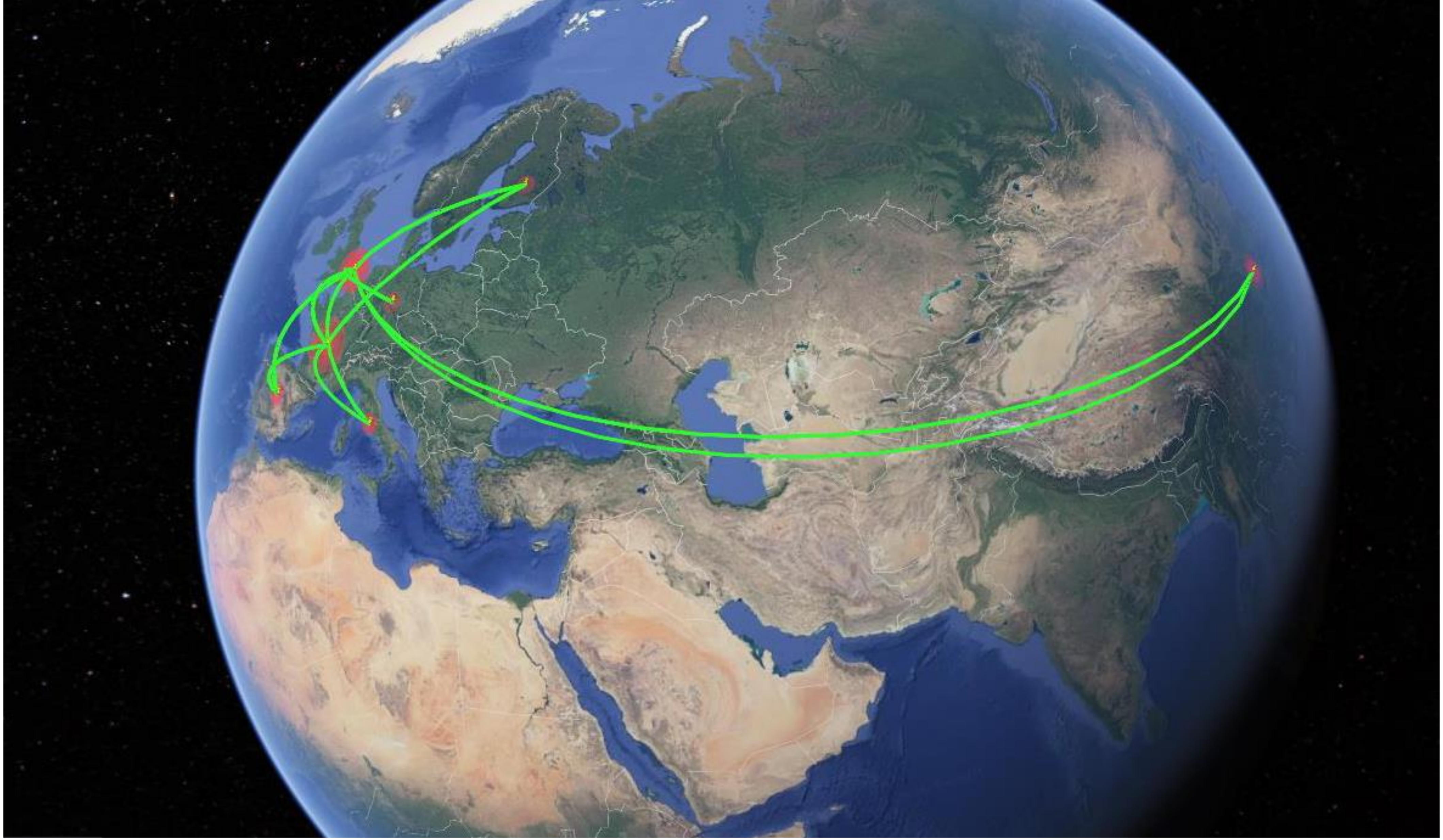

Supplementary File S1. Spatiotemporal spread of SARS-CoV-2 in Europe. The MCC of the dataset including sequences from Wuhan and from the first European countries reporting positive cases was projected on a map using SpreaD3 and can be visualized with a virtual globe software like Google Earth.
